# A mechanistic study of *Toxoplasma gondii* ROP18 inhibiting differentiation of C17.2 neural stem cells

**DOI:** 10.1186/s13071-017-2529-2

**Published:** 2017-11-23

**Authors:** Xian Zhang, Rui Su, Zhengyang Cheng, Wanbo Zhu, Yelin Li, Yongzhong Wang, Jian Du, Yihong Cai, Qingli Luo, Jilong Shen, Li Yu

**Affiliations:** 1 Department of Microbiology and Parasitology; Anhui Provincial Laboratory of Microbiology and Parasitology; Anhui Key Laboratory of Zoonoses, School of Basic Medical Sciences, Anhui Medical University, Hefei, 230032 People’s Republic of China; 2 Anhui Key Laboratory of Modern Biomanufacturing, School of Life Sciences, Anhui University, Hefei, 230039 People’s Republic of China; 3 Department of Biochemistry, Anhui Medical University, Hefei, 230032 People’s Republic of China; 4 Department of Health Inspection and Quarantine, School of Public Health, Anhui Medical University, Hefei, China

**Keywords:** *T. gondii*, Neural stem cells, C17.2, Differentiation, ROP18

## Abstract

**Background:**

Congenital infection of *Toxoplasma gondii* is an important factor causing birth defects. The neural stem cells (NSCs) are found to be one of the target cells for the parasite during development of the brain. As a key virulence factor of the parasite that hijacks host cellular functions, ROP18 has been demonstrated to mediate the inhibition of host innate and adaptive immune responses through specific binding different host immunity related molecules. However, its pathogenic actions in NSCs remain elusive.

**Results:**

In the present study, ROP18 recombinant adenovirus (Ad-ROP18) was constructed and used to infect C17.2 NSCs. After 3d- or 5d–culture in differentiation medium, the differentiation of C17.2 NSCs and the activity of the Wnt/β-catenin signaling pathway were detected. The results showed that the protein level of βIII-tubulin, a marker of neurons, in the Ad-ROP18-transfected C17.2 NSCs was significantly decreased, indicating that the differentiation of C17.2 NSCs was inhibited by the ROP18. The β-catenin level in the Ad-ROP18-transfected C17.2 NSCs was found to be lower than that in the Ad group. Also, neurogenin1 (Ngn1) and neurogenin2 (Ngn2) were downregulated significantly (*P* < 0.05) in the Ad-ROP18-transfected C17.2 NSCs compared to the Ad group. Accordingly, the TOP flash/FOP flash dual-luciferase report system showed that the transfection of Ad-ROP18 decreased the Wnt/β-catenin pathway activity in the C17.2 NSCs.

**Conclusions:**

The inhibition effect of the ROP18 from *T. gondii* (TgROP18) on the neuronal differentiation of C17.2 NSCs was at least partly mediated through inhibiting the activity of the Wnt/β-catenin signaling pathway, eventually resulting in the downregulation of Ngn1 and Ngn2. The findings help to better understand potential mechanisms of brain pathology induced by TgROP18.

## Background

*Toxoplasma gondii* is arguably the most successful obligate intracellular parasite. It infects a variety of warm-blooded vertebrates once the infectious oocysts or tissue cysts from contaminated food or water are ingested orally. It is reported that around one third of the worldwide population is infected by the parasite with varying infection rates among different countries [1–3]. Of those who are infected, typically most show minor or no apparent symptoms owing to their strong immune controls. However, for pregnant women and individuals who have compromised immune systems, such as AIDS, cancer chemotherapy and transplantation, *Toxoplasma* infection could cause serious health problems. Among the outcomes of the parasitic infection, congenital toxoplasmosis has been reported as the most serious with an incidence of around 1–15 per 10,000 live births [4]. Typically, congenital toxoplasmosis shows different symptoms, including hydrocephalus or microcephalus, intracerebral calcification and chorioretinitis, etc. [5]. *Toxoplasma gondii* has the capability to invade almost any nucleated cells and any organ; however, the brain is known to be the most easily damaged site after the infection with this parasite. Presumably, the neural stem cell (NSCs) should be one of the target cells for the infection during the development of the brain and neuropathogenesis. However, the exact mechanisms of pathological brain damage in congenital toxoplasmosis still remain unknown.

*Toxoplasma gondii* is a model apicoplexan, with a characteristic apical complex. This apical complex is composed of specialized cytoskeletal and several secretory organelles. Rhoptry (ROP) is one of the typical secretory organelles, by which the effector proteins of rhoptry are secreted. The secreted ROP proteins are known to play an important role in active penetration of the parasite into the host cell and eventually manipulate the host cell towards their needs [6, 7]. Among these secreted ROP proteins, ROP18 has been regarded as a key virulence factor that can hijack cellular functions of the host cells [8, 9]. It is highly expressed in types I and II strains of *T. gondii*, and targets the parasitophorous vacuole membrane (PVM) following its secretion. Similar to other members of the ROP2 clade, ROP18 is also composed of the conserved residues that are required for the activity of S/T kinase, and it has been reported to phosphorylate other ROP proteins (i.e. ROP2, ROP4 and ROP8) and interacts with other host cell proteins. For example, it targets immunity-related GTPases (IRGs) by activating transcript factor 6β (ATF6β), and p65 to inhibit host innate and adaptive immune responses [10–14]. Despite the mechanism of ROP18-mediated virulence through escaping from immune system eliminations is well studied, the exact molecular mechanisms of this kinase exerting its pathogenic action in NSCs remain poorly understood.

Congenital infection of pathogens can induce abnormal proliferation, differentiation or apoptosis of the neural stem cells (NSCs), eventually causing brain malformations [15–17]. Our previous studies demonstrated that both *Toxoplasma* RH strain (a canonical type I strain) and TgCtwh3 strain (a representative Chinese 1 strain) were able to infect the GD14 embryos of ICR (Institute of Cancer Research) mice-derived NSCs and the cultured C17.2 NSCs, leading to apparent apoptosis of these NSCs. These findings may partly explain the possible mechanisms of pathological brain damage caused by congenital *T. gondii* infection [18, 19]. Furthermore, we recently found that the excreted-secreted antigens (ESAs) of *T. gondii* RH inactivated the differentiation of C17.2 NSCs and downregulated the expression level of β-catenin [20]. β-catenin is a crucial component of the Wnt/β-catenin signaling pathway. Activity of the Wnt/β-catenin signaling pathway is modulated by the interactions of β-catenin and many protein kinases (e.g. GSK-3b, CK1a) [21]. As an important virulent factor, we speculate that ROP18 secreted by *T. gondii* secretory organelles (TgROP18) has an inhibition effect on the differentiation of NSCs. To understand the exact molecular mechanisms of the inhibition effect on the NSCs differentiation induced by TgROP18, we transfected C17.2 NSCs with TgROP18 recombinant adenovirus (Ad-ROP18), and then detected the differentiation of C17.2 NSCs and the activity of the Wnt/β-catenin signaling pathway. Our data revealed that ROP18 kinase exerts the inhibition effect on the neuronal differentiation of NSCs via inhibiting the activity of the Wnt/β-catenin signaling pathway. ROP18 has been demonstrated to mediate the inhibition of host innate and adaptive immune responses through specific binding different host immunity related molecules; however, its pathogenic actions in NSCs remain elusive. The findings would help us to better understand the potential mechanisms of pathological brain damage induced by *T. gondii.*

## Methods

### Cell culture

C17.2 NSCs, a murine neural stem cell line, were donated by Dr Evan Y. Snyder from Burnham Institute for Medical Research (La Jolla, CA, USA), and were cultured following the standard operating procedure [22, 23]. Briefly, the cells were grown in a 5% CO_2_ incubator at 37 °C using DMEM complete medium plus 10% fetal bovine serum, 5% horse serum (Gibco, Grand Island, USA), 2 mM L-glutamine (Gibco), 100 μg/ml streptomycin (Sigma-Aldrich, St. Louis, USA), and 100 μg/ml penicillin (Sigma-Aldrich). The cell monolayers were detached by 0.05% trypsin-EDTA and reseeded when they reached 70–80% confluence. The suspended cells were split no more than 1:10 once per week.

### Differentiation of C17.2 NSCs

The C17.2 NSCs (~4 × 10^5^) were reseeded onto 12 mm matrigel-coated coverslips in 60 mm Petri dishes; uncoated coverslips were used as controls. The cells were then cultured for 24 h in DMEM complete medium plus 5% horse serum and 10% fetal bovine serum. The medium was then changed to serum-free DMEM/F12 plus 2% N2 when the cell monolayers reached 50% confluence to induce the differentiation of the C17.2 NSCs. The morphological changes in the C17.2 NSCs were recorded every day. The cells were subject to immunofluorescence staining of βIII-tubulin on day 3 and 5 to evaluate the differentiation level.

### Construction and identification of TgROP18 recombinant adenovirus

With the template pEGFP-G2-ROP18, PCR was conducted with specific primers for TgROP18 CDS (Rop18-F: 5′-ATT AGC GGC CGC ATG TTT TCG GTA CAG CGG CCA-3′; Rop18-R: 5′-ACA CAT GCA TTT ATT CTG TGT GGA GAT G-3′). The forward and reverse primers introduced 5′ terminal restriction sites of *Not*I and *Nsi*I, respectively. PCR was performed as follows: preheating at 94 °C for 5 min, then 30 cycles of 94 °C for 30 s min, 55 °C for 30 s, and 72 °C for 90 s, plus a final extension at 72 °C for 10 min. Electrophoresis of the PCR products was conducted on 1.5% agarose gel stained with ethidium bromide, and then the PCR segments were recovered and purified according to the DNA Pure-Spin Kit (Axgen, Tewksbury, USA). Purified TgROP18 DNA segment and pHBAd-MCMV-GFP vector (Hanbio, Shanghai, China) were digested with restriction endonuclease *Not*I and *Nsi*I, respectively. Recovered products were ligated with T4 DNA ligase, and transformed into chemically competent *E. coli* DH5α cells. The positive transformants were selected on Amp-LB agar plates, and then inoculated in LB liquid culture media. After overnight incubation, the recombinant plasmid DNA was isolated by alkaline lysis method and further confirmed via *Not*I and *Nsi*I double digestion [24]. pHBAd-MCMV-GFP-Rop18 was sequenced by Shanghai Sunny Biotechnology Co., Ltd. (China), and the resultant sequence was identical to TgROP18 gene in the GenBank database (accession: AM075204).

HEK 293 cells were seeded at a density of 1.5 × 10^6^ cells per 60 mm Petri dish, and then cultured for 24 h before transfection. 2 μg of pHBAd-MCMV-GFP-ROP18 vector and 4 μg of adenoviral backbone plasmid pHBAd-BHG (Hanbio, Shanghai, China) were transfected into 293 cells with Lipofiter™ (Sunny Biotechnology Co. Ltd., Shanghai, China) according to the transfection agent manual. Two to three days later, the adherent cells with obvious cytopathic effect (CPE) became rounded and ablated. The collected cells were repeatedly freeze-thawed at least three times at 37 °C/-70 °C. The Ad-ROP18 virus-containing supernatant was obtained after centrifugation at 3000× *rpm*, and then the titer determined with an improved TCID50 method [25].

### Transfection of C17.2 NSCs with ad-ROP18

The C17.2 NSCs were cultured in DMEM complete medium plus 5% horse serum and 10% fetal bovine serum following reseeding at a density of 1 × 10^5^ cells/ml in 6-well plates. After 24 h incubation, the cell monolayers were washed with phosphate buffered saline (PBS) three times, and then the Ad-ROP18 or blank recombinant adenovirus (Ad) at multiplicity of infection (MOI) of 70 was transfected into C17.2 NSCs and cultured in serum-free DMEM/F12 plus 2% N2 in a 5% CO_2_ air incubator at 37 °C for 3 or 5 days. The expression of ROP18 in C17.2 NSCs was analyzed by western blotting and fluorescence microscopy (Olympus, Tokyo, Japan).

### Immunofluorescence

The C17.2 cells were grown on coverslips in 5% CO_2_ at 37 °C for 3 or 5 days. After the cells were washed twice with PBS, they were fixed with 4% formaldehyde for 20 min, then washed three times with PBS, permeabilized with 0.1% TritonX-100 for 15–20 min, and finally blocked for 1 h with 5% bovine serum albumin in PBS. The cells were then subject to incubation with mouse anti-βIII tubulin monoclonal antibody (1:200, Cell Signaling Technology, USA) overnight at 4 °C, and then incubated for 1 h at 37 °C; finally, they were washed with PBS three times. The coverslips were then subjected to incubation with PE-conjugated anti-mouse IgG (1:200 dilution; Santa Cruz, USA) at 37 °C for 1 h. After that, the cells were washed with PBS for three times, and stained with Hoechst 33,258 (Sigma-Aldrich) for 10 min at room temperature to observe the nucleus. The images were recorded using fluorescent microscopy (Olympus).

### Western blotting

The expression levels of ROP 18, βIII-tubulin, neurogenin1 (Ngn1), neurogenin2 (Ngn2) and β-catenin were determined by western blotting to further identify the differentiation of C17.2 NSCs. Briefly, after the C17.2 NSCs were transfected with Ad-ROP18 or Ad and then cultured for 3 days and 5 days, the cells were harvested and total proteins were isolated. 40 μg of total proteins were electrophoresed on 12% sodium dodecyl sulfatepolyacrylamide gel (SDS-PAGE), and electrically transferred onto a nitrocellulose membrane (Millipore, Billerica, USA), and then immunoblotted using primary monoclonal antibodies (Cell Signaling Technology, Danvers, USA): anti-βIII tubulin antibody (1:1000), anti-Ngn1 antibody (1:500), anti-Ngn2 antibody (1:1000), anti-β-catenin antibody (1:1000) and anti-β-actin antibody (1:1000). β-actin, in this study, was used as a loading control. Blots were subsequently incubated for 2 h at room temperature with respective to secondary antibodies, including anti-mouse (for anti-βIII tubulin and anti-Ngn1 primary antibodies) and anti-rabbit IgG (for anti-Ngn2, anti-β-catenin and anti-β-actin primary antibodies) conjugated with horseradish peroxidase (1:5000, ZSGB-Bio, Beijing, China). An ECL kit (Thermo Scientific, Waltham, USA) was used to detect the chemiluminescence. The results were analyzed by using JD-801 gel imaging analysis system (Panasonic, Osaka, Japan).

### Luciferase reporter assay

The TOP flash/FOP flash dual-luciferase report system was used to detect Wnt signaling pathway activity of the Ad-ROP18- or Ad-transfected C17.2 NSCs. Briefly, C17.2 NSCs were plated at a density of 1 × 10^4^ cells/ml in 24-well plates. After 16 h culture, 500 ng TOP flash plasmid (Millipore) containing six TCF-binding motifs and firefly luciferase open reading frame was used to transfect the cells in Opti-MEM using lipofectamine 2000 (Invitrogen) according to the manufacturer’s protocol. Meanwhile, 500 ng FOP flash plasmid containing six mutated TCF-binding motifs (Millipore), and 10 ng pRL-SV40 vector containing renilla luciferase reporter gene (Promega, Madison,USA) were used to normalize the transfection efficiency. After 6 h culture, serum-free DMEM/F12 plus 2% N2 was used to replace the Opti-MEM, followed by infection of Ad-ROP18 or Ad at MOI of 70. After 3d- or 5d-incubation, the luciferase activity was evaluated according to Dual-Luciferase Reporter (DLR™) Assay System (Promega). The cells were incubated with passive lysis buffer (PLB) for 15–20 min on an orbital shaker to lyse the cells. The lysates were spun down at 12,000× *rpm* for 1 min at 4 °C, and the supernatants were collected. The cell lysates were placed into a luciferase assay reagent II-containing luminometer tube, and were mixed by pipetting 2 or 3 times. After measurement of firefly luciferase (Fluc), the signals were quenched by adding stop & glo reagent, followed by the reaction with renilla luciferase (Rluc) substrate. The signals in arbitrary unit (AU) from both Rluc and Fluc were measured by a luminometer (Promega, GloMax 20/20, Madison, USA). Normalization of the relative firefly luciferase activity was conducted using the renilla luciferase activity.

### Statistical analysis

All quantitative data were represented as mean ± standard error of mean (SEM). The differences between two groups were analyzed by Student’s *t*-test and *P* < 0.05 was defined as a statistically significant difference.

## Results

### Expression of ad-ROP18 in C17.2 NSCs

**Fig. 1:**
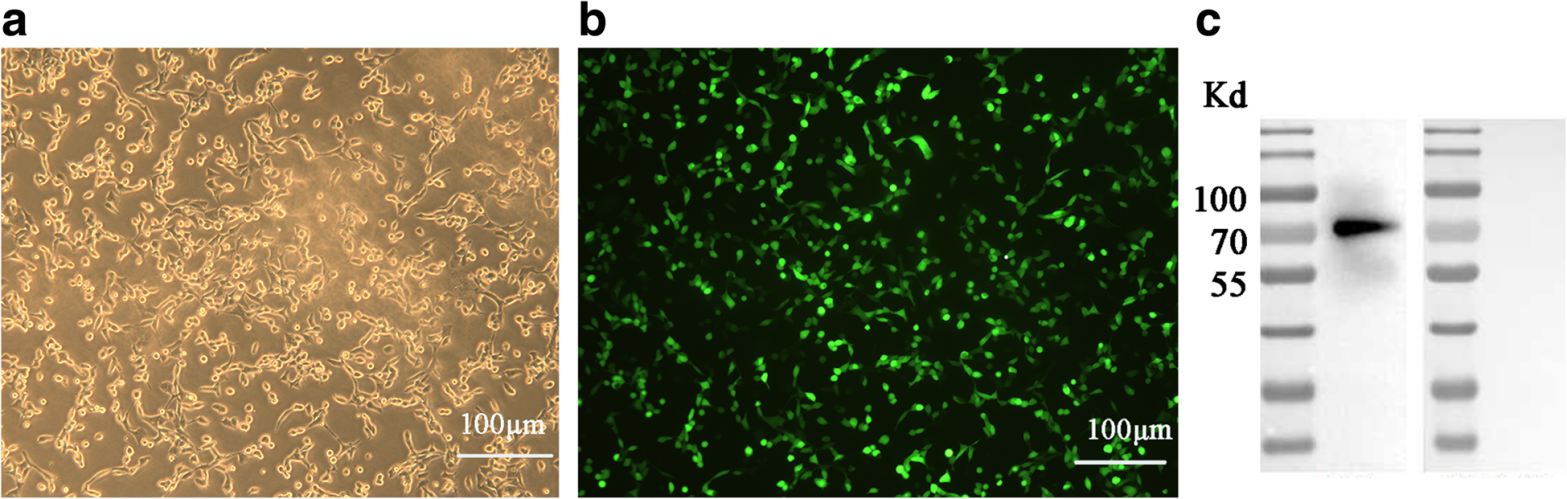
Expression of Ad-ROP18 in C17.2 NSCs. The bright field image of the C17.2 NSCs after transfected with Ad-ROP18 (**a**). The fluorescent detection of C17.2 NSCs (**b**) and western blotting of C17.2 NSCs (**c**) after the cells transfected with Ad-ROP18

After digestion with *Not*I and *Nsi*I, ROP18 was ligated to the pHBAd-MCMV-GFP vector. After 24 h incubation, the cultured C17.2 NSCs were transfected by Ad-ROP18 at MOI of 70, and the blank recombinant adenovirus, i.e. Ad, was used as a control. To calculate the percentage of cells expressing ROP18, we randomly selected 5 fields under a microscope and counted the number of cells with or without green fluorescence. As shown in Fig. 1, the fluorescent signal was recorded after C17.2 NSCs were transfected by Ad-ROP18, and the percentage of cells with green fluorescence was about 90%. Additionally, fusion protein of ROP18-GFP was successfully determined by western blotting assay using the ROP18 polyclonal antibody, and an expected 70 KD band was found. The results indicated that ROP18 recombinant adenovirus was highly expressed in the C17.2 NSCs.

### The differentiation of ad-ROP18-transfected C17.2 NSCs

**Fig. 2:**
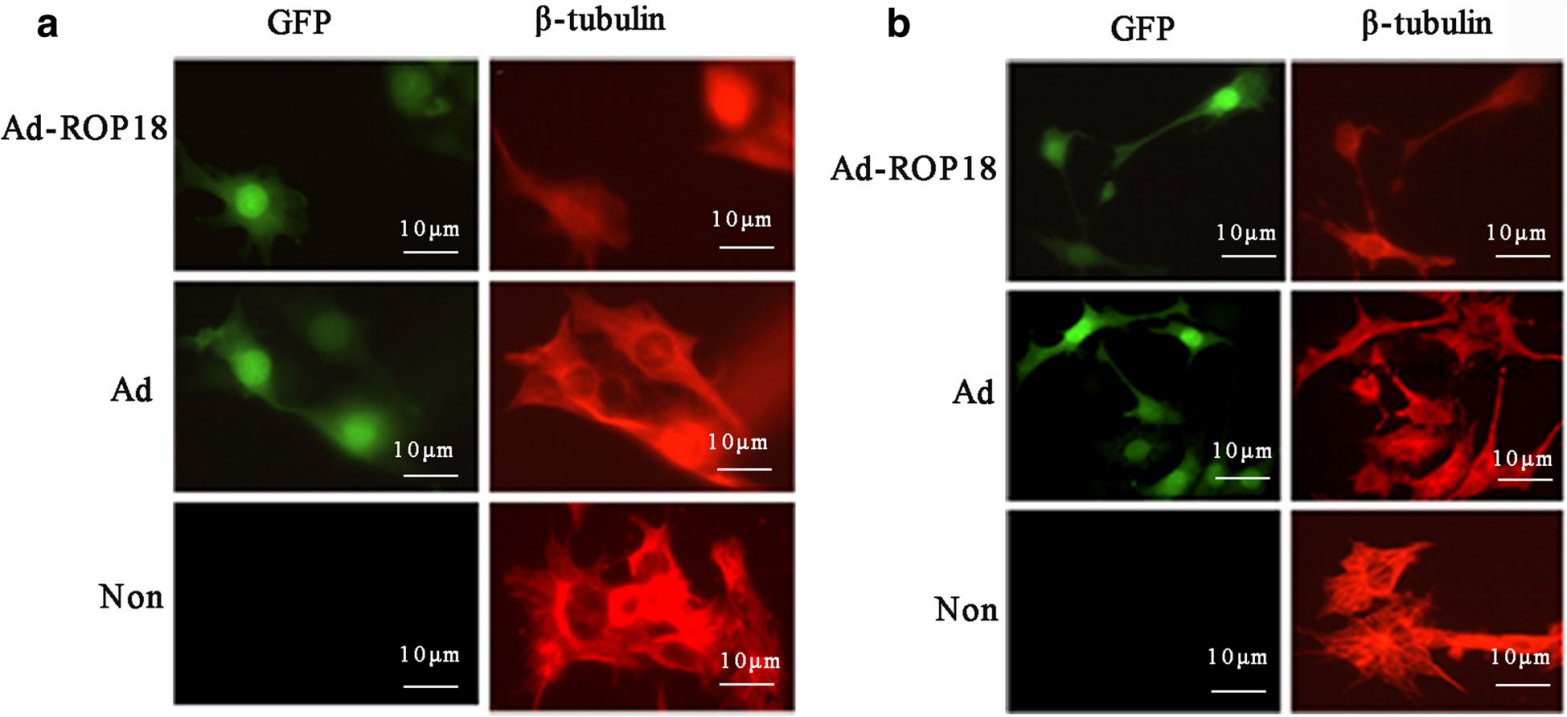
Immunofluorescence staining of βIII-tubulin. C17.2 NSCs were infected with or without Ad-ROP18 at MOI of 70, and then cultured in DMEM: F12 supplemented with 2% N2 for 3 days (**a**) or 5 days (**b**). The expression of βIII-tubulin in the cells were identified using immunofluorescence staining (green: GFP; red: βIII-tubulin). *Abbreviations*: Non, non-infection group, C17.2 NSCs without Ad-ROP18 infection; Ad, C17.2 NSCs infected with adenovirus empty vector; Ad-ROP18, C17.2 NSCs infected with Ad-ROP18 recombinant adenovirus

To evaluate the effect of Ad-ROP18 on the differentiation of C17.2 NSCs, the expression levels of βIII-tubulin in the cells infected with or without Ad-ROP18 at an MOI of 70 were detected by immunofluorescence staining. Ten fields were selected randomly and staining signals in the cytoplasm were examined by visual observation. As shown in Fig. 2, the βIII-tubulin staining in the C17.2 NSCs cultured in DMEM/F12 plus 2% N2 was obvious on both days 3 and 5. However, the staining signals in the Ad-ROP18-infected cells were significantly decreased.

**Fig. 3:**
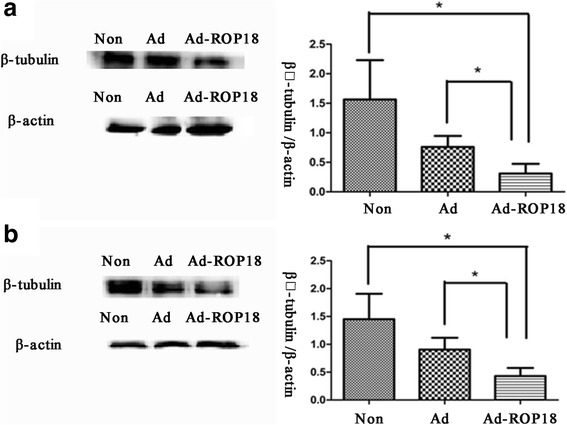
The relative protein levels of βIII-tubulin in C17.2 NSCs were detected by western blotting. C17.2 NSCs were infected with/without Ad-ROP18 at MOI of 70 and then cultured in DMEM: F12 supplemented with 2% N2 for 3 days (**a**) or 5 days (**b**). The examinations are repeated three times. The resented figures are from a representative analysis, and all these data represent the mean ± SD on different assays (*n* = 3), *P < 0.05. *Abbreviations*: Non, non-infection group, C17.2 NSCs without Ad-ROP18 infection; Ad, C17.2 NSCs infected with adenovirus empty vector; Ad-ROP18, C17.2 NSCs infected with Ad-ROP18 recombinant adenovirus

After immunofluorescence staining, the protein levels of βIII-tubulin in the C17.2 NSCs were assessed by western blotting. As shown in Fig. 3, the relative levels of βIII-tubulin in the Ad-ROP18-transfected C17.2 NSCs were 0.3100 ± 0.0945 for day 3 and 0.4297 ± 0.0845 for day 5. These levels were significantly lower than those in the non-infected C17.2 NSCs grown in DMEM/F12 plus 2% N2 1.563 ± 0.3846 for day 3 (*t*_(4)_ = 3.165, *P* = 0.034) and 1.452 ± 0.2628 for day 5 (*t*_(4)_ = 3.702 *P* = 0.021)], and also significantly lower than those in the Ad-transfected C17.2 NSCs [0.7597 ± 0.1081 for day 3 (*t*_(4)_ = 3.312, *P* = 0.035) and 0.9057 ± 0.1222 for day 5 (*t*_(4)_ = 3.204, *P* = 0.032)].

### Expression of β-catenin and neurogenin in ad-ROP18-transfected C17.2 NSCs

**Fig. 4:**
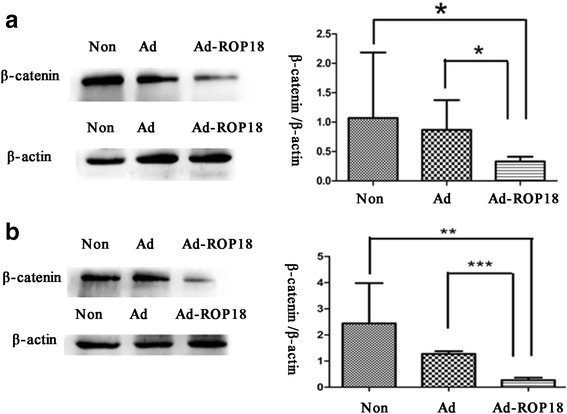
The protein levels of β-catenin in C17.2 NSCs.C17.2 NSCs were infected with or without Ad-ROP18 at MOI of 70, and then were cultured in DMEM: F12 supplemented with 2% N2 for 3 days (**a**) or 5 days (**b**). The protein levels of β-catenin and β-actin were detected by western blotting. The examinations are repeated three times. The presented figures are from a representative analysis, and all these data represent the mean ± SD on different assays (*n* = 3), *P < 0.05. *Abbreviations*: Non, non-infection group, C17.2 NSCs without Ad-ROP18 infection; Ad, C17.2 NSCs infected with adenovirus empty vector; Ad-ROP18, C17.2 NSCs infected with Ad-ROP18 recombinant adenovirus

In order to examine whether ROP18 inhibits the differentiation of C17.2 NSCs via the Wnt/β-catenin signaling pathway, the protein level of β-catenin in the Ad-ROP18-transfected cells was first detected. As shown in Fig. 4a, after 3 d-culture, the β-catenin level in Ad-ROP18-transfected C17.2 NSCs was 0.3277 ± 0.1294, significantly lower than that in the Ad-transfected cells (0.8263 ± 0.1096) (*t*_(4)_ = 2.94, *P* = 0.042), and greatly lower than that in the non-transfected cells cultured in the same differentiation medium (2.363 ± 1.201). As shown in Fig. 4b, on day 5, the protein levels of β-catenin in the Ad-ROP18-tansfected C17.2 NSCs were also significantly decreased as compared to the Ad-transfected or non-transfected cells. The protein levels of β-catenin in the Ad-ROP18-transfected, Ad-transfected, and non-transfected C17.2 NSCs were 0.2700 ± 0.0538, 1.273 ± 0.0602 and 2.440 ± 0.8909, respectively.

**Fig. 5:**
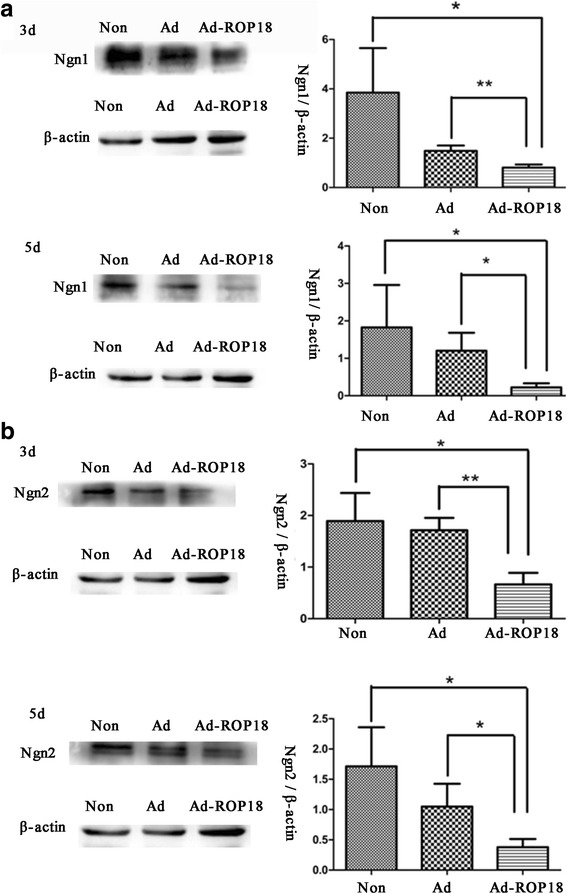
The expression of Ngn1 and Ngn2 in C17.2 NSCs. C17.2 NSCs were infected with or without Ad-ROP18 at MOI of 70, and then were cultured in DMEM: F12 supplemented with 2% N2 for 3 or 5 days. The protein levels of Ngn1 (**a**), Ngn2 (**b**) were detected by western blotting. The examinations are repeated three times. The presented figures are from a representative analysis, and all these data represent the mean ± SD on different assays (*n* = 3), *P < 0.05. *Abbreviations*: Non: non-infection group, C17.2 NSCs without Ad-ROP18/Ad infection; Ad: C17.2 NSCs infected with adenovirus empty vector; Ad-ROP18: C17.2 NSCs infected with Ad-ROP18 recombinant adenovirus

Next, the protein levels of neurogenin Ngn1 and Ngn2, the neuron-specific transcription factors, in the Wnt/β-catenin signaling pathway in C17.2 NSCs were detected after being treated with or without Ad-ROP18 for 3 or 5 days. As shown in Fig. 5, the expression of Ngn1 in C17.2 NSCs after treatment with Ad-ROP18 for 3 days (0.8073 ± 0.0700) was inhibited, significantly lower than that in the Ad group (1.485 ± 0.1259) (*t*_(4)_ = 4.647, *P* = 0.009) and the non-infection group (3.848 ± 1.042) (*t*_(4)_ = 2.901, *P* = 0.044). On day 5, the expression of Ngn1 in C17.2 NSCs treated with Ad-ROP18 (0.2213 ± 0.0633) was significantly decreased as compared to that in the Ad group (1.202 ± 0.2765) (*t*_(4)_ = 3.456, *P* = 0.026). A similar expression profile was also found for Ngn2 after treatment of the cells with Ad-ROP18 for 3 days and 5 days. On day 3, the protein levels of Ngn2 in C17.2 NSCs for Ad-ROP18 group, Ad group and non-infection group were 0.6627 ± 0.1309, 1.713 ± 0.1396, and 1.890 ± 0.3145, respectively. On day 5, the levels were 0.3777 ± 0.0783, 1.050 ± 0.2177 and 1.714 ± 0.3725, respectively. Both on day 3 and 5, the Ngn2 level in the C17.2 NSCs treated with Ad-ROP18 was significantly decreased compared with those in the Ad group or the non-infection group (*t*_(4)_ = 3.512, *P* = 0.025).

### Activity of the Wnt/β-catenin signaling pathway in ad-ROP18-transfected C17.2 NSCs

**Fig. 6:**
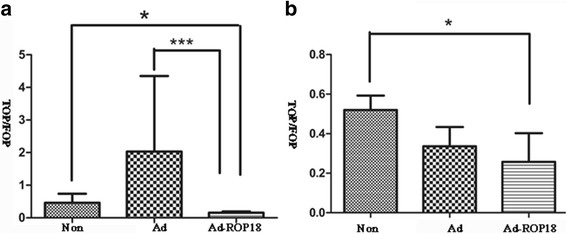
Activity of the Wnt/β-catenin signaling pathway in C17.2 NSCs detected by TOP flash/FOP flash dual-luciferase report system. C17.2 NSCs were transfected with TOP flash/ FOP flash plasmid and pRL-SV40 vector, followed by infection with or without Ad-ROP18 at MOI of 70, and then were cultured in DMEM: F12 supplemented with 2% N2 for 3 or 5 days. The activities of firefly luciferase and renilla luciferase were measured by a luminometer, and the activity of the Wnt/β-catenin signaling pathway was recorded as TOP/FOP. All these data represent the mean ± SD on different assays (*n* = 3), *P < 0.05. *Abbreviations*: Non, non-infection group, C17.2 NSCs without Ad-ROP18/Ad infection; Ad, C17.2 NSCs infected with adenovirus empty vector; Ad-ROP18, C17.2 NSCs infected with Ad-ROP18 recombinant adenovirus

To further test the activity of the Wnt/β-catenin signaling pathway, a TOP falsh/FOP flash dual-luciferase report system was employed. TOP/FOP luciferase activities in C17.2 NSCs treated with Ad-ROP18 greatly lower (0.1553 ± 0.0234) than that in the Ad group (2.029 ± 1.339) or the non-infection group (0.4618 ± 0.1614) on day 3 (Fig. 6). They were significantly lower (*t*_(4)_ = 2.794, *P* = 0.049) than that in non-infection group on day 5. However, no significant differences were found between the Ad-ROP18 group and the Ad group on day 5 *t*_(4)_ = 0.782, *P* = 0.478).

## Discussion

Since Boothroyd’s and Sibley’s laboratories identified ROP18 as a critical contributor to virulence of the parasite using classical forward genetic approaches, many studies have focused on the molecular mechanisms of TgROP18 manipulating host cellular functions [8, 9]. It is reported that TgROP18 could inhibit innate immunity through downregulation of IFN-γ-inducible GTPases and subsequent prevention from targeting PVM in mice [13, 14, 26, 27]. In addition, it can also inhibit adaptive immunity by targeting activated transcript factor 6β (ATF6β), a component of the unfolded protein response [10, 11], and by phosphorylating the host p65, a family member of NF-κB [12], to downregulate type I immune responses mediated by CD8 T cells. *Toxoplasma gondii* is a neurotropic parasite, and can infect many kinds of cells in the central nervous system, including NSCs. However, the molecular mechanism of this virulent protein exerting neuropathogenesis remains poorly understood. Our recent studies examined the effect of TgROP18 on the apoptosis of neural cells and demonstrated that TgROP18 could induce the ER stress-mediated apoptosis pathway to facilitate neural cell death [28, 29]. As the number of differentiated cells and the size of each region of the brain are dependent on the balance and fate decision between proliferation and differentiation of the NSCs, in this study we further evaluated the effect of TgROP18 on the differentiation of NSCs, a key process of brain development and our data have shown that it inhibited the neuronal differentiation of C17.2 NSCs. Together, these data reveal that TgROP18 is a crucial factor that relevantly induces pathological brain damage, though the molecular mechanism of inhibition of this kinase on the central nervous system remains to be explored.

In this study, we explored the inhibition effect of TgROP18 on the Wnt signaling pathway in the C17.2 NSCs. Since the Wnt signaling pathway was reported to play a crucial role in the development of the brain and the differentiation of NSCs [21, 30, 31], the inhibition effect of TgROP18 on the Wnt pathway in the C17.2 NSCs was reasonably proposed and subsequently investigated by determining the protein levels and activities of the related proteins involved in this pathway. Wnt signaling pathways were categorized into canonical and noncanonical Wnt pathways, dependent on their requirement or independence of intracellular β-catenin. In the canonical Wnt pathway, also known as Wnt/β-catenin pathway, a degradation complex, consisting of Axin, casein kinase 1 (CK1), glucogen synthase kinase 3β (GSK3β) and adenomatous polyposis coli (APC), keeps the central co-activator β-catenin at very low levels. This cytoplasmic degradation complex allows the phosphorylation of β-catenin, and sequential polyubiquitination by the E3 ligase β-TrCP, and final degradation mediated by the proteasome [21]. In an active state, once Wnt proteins bind to the co-receptors and receptors on the cell surface, the formation of the degradation complex is inhibited, leading to the stabilization and accumulation of β-catenin in cytoplasma. Eventually, the stabilized β-catenin in the cytoplasma is translocated into the nucleus to act as a transcriptional coactivator of several transcription factors in the TCF/LEF family. Thus, β-catenin plays a pivotal role in the control of decisive steps in the development of the brain through the canonical Wnt signaling system. It has been previously reported that conditional mutation of β-catenin could cause the elimination of the cells at the mid-hindbrain boundary, and decrease the neuronal precursor population and overall size of the nervous system [32, 33]. In this study, we examined the protein levels of β-catenin in the TgROP18-treated C17.2 NSCs. The data revealed that the β-catenin level in the C17.2 NSCs was significantly decreased after TgROP18 treatment for 3 or 5 days, indicating that the Wnt/β-catenin signaling pathway was inactivated by theTgROP18 transfection in the C17.2 NSCs. To further examine the effects of TgROP18 on the activity of the Wnt/β-catenin signaling pathway, the TOP flash/FOP flash assay dual-luciferase report system was used. Consistent with a decrease in β-catenin level, significantly lower activities of the TOP/FOP luciferase were found in the TgROP18-transfected C17.2 NSCs, as compared to the control cells, indicating that TgROP18 could attenuate β-catenin/TCF transcriptional activity in the C17.2 NSCs.

On the question of how TgROP18 inhibits neuronal differentiation via the Wnt/β-catenin pathway in NSCs, a previous study found that a nucleotide sequence at positions -1167 to -1160 in the Ngn1 gene promoter, called TCF binding element, was necessary for maximal transcriptional activity of the Ngn1 gene in neural progenitor cells (NPCs), and β-catenin was able to directly bind the promoter to enhance the level of Ngn1 [34]. An ectopic expression of stabilized β-catenin was demonstrated to increase the mRNA level of Ngn1 [34]. More importantly, a reduced expression of Ngn1 was also found in β-catenin-deficient neural crest cells [31]. These results show that β-catenin can mediate the regulation of Ngn1. As is well known, Ngn1, as a proneural basic helix-loop-helix (bHLH) transcription factor [35], together with Ngn2, both were expressed in immature neurons and newly committed neuronal progenitors, playing crucial roles in neurogenesis and regional specification in the neocortex [36]. In this study, the expression level of Ngn1 in NSCs treated with TgROP18 was detected, and we found that the expression level of Ngn1 in the TgROP18-treated NSCs was significantly decreased, which was consistent with the expression profile of β-catenin. The results imply that TgROP18 inhibits the neuronal differentiation of NSCs through the Wnt/β-catenin pathway by regulation of Ngn1 expression. Since Notch signaling is also involved in the neurogenesis, playing an antagonistic interaction with the Wnt pathways, how the Notch and the Wnt pathways interact in the C17.2 NSCs after treatment with TgROP18 needs to be further explored.

## Conclusions

Our study shows that the TgROP18 inhibits the neuronal differentiation of the C17.2 NSCs. This effect is mediated at least partly by inhibiting the activity of the Wnt/β-catenin signaling pathway, subsequently resulting in the downregulation of Ngn1 and Ngn2. These findings reveal a potential molecular mechanism of pathological brain damage induced by TgROP18.

## Abbreviations

APC: Adenomatous polyposis coli
bHLH: Basic helix-loop-helix
CK1: Casein kinase 1
ESAs: Excreted-secreted antigens
GSK3β: Glucogen synthase kinase 3β
IRGs: Immunity-related GTPases
Ngn1: Neurogenin1
Ngn2: Neurogenin2
NPCs: Neural progenitor cells
NSCs: Neural stem cells
PVM: Parasitophorous vacuole membrane
ROPs: Proteins of rhoptries

## References

[1] Tenter AM, Heckeroth AR, Weiss LM (2000). *Toxoplasma gondii*: from animals to humans. Int J Parasitol.

[2] Dubey JP (2010). Toxoplasmosis of animals and humans.

[3] Hill D, Dubey JP (2002). *Toxoplasma gondii*: transmission, diagnosis and prevention. Clin Microbiol Infect.

[4] Andreoletti O, Budka H, Buncic S, Colin P, Collins DJ, De Koeijer A (2007). Scientific opinion of the panel on biological hazards on a request from EFSA on surveillance and monitoring of *Toxoplasma* in humans, foods and animals. EFSA J.

[5] Petersen E (2007). Toxoplasmosis. Semin Fetal Neonatal Med.

[6] Bradley PJ, Sibley LD (2007). Rhoptries: an arsenal of secreted virulence factors. Curr Opin Microbiol.

[7] Boothroyd JC, Dubremetz JF (2008). Kiss and spit: the dual roles of *Toxoplasma* rhoptries. Nat Rev Microbiol.

[8] Saeij JP, Boyle JP, Coller S, Taylor S, Sibley LD, Brooke-Powell ET (2006). Polymorphic secreted kinases are key virulence factors in toxoplasmosis. Science.

[9] Taylor S, Barragan A, Su C, Fux B, Fentress SJ, Tang K, et al. A secreted serine-threonine kinase determines virulence in the eukaryotic pathogen *Toxoplasma gondii*. Science. 2006;314(5806):1776–80. 10.1126/science.1133643 17170305

[10] Yamamoto M, Ma JS, Mueller C, Kamiyama N, Saiga H, Kubo E (2011). ATF6beta is a host cellular target of the *Toxoplasma gondii* virulence factor ROP18. J Exp Med.

[11] Yamamoto M, Takeda K. Inhibition of ATF6beta-dependent host adaptive immune response by a *Toxoplasma* virulence factor ROP18. Virulence. 2012;3(1):77–80. 10.4161/viru.3.1.18340 22286708

[12] Du J, An R, Chen L, Shen Y, Chen Y, Cheng L (2014). *Toxoplasma gondii* virulence factor ROP18 inhibits the host NF-kappaB pathway by promoting p65 degradation. J Biol Chem.

[13] Fentress SJ, Behnke MS, Dunay IR, Mashayekhi M, Rommereim LM, Fox BA (2010). Phosphorylation of immunity-related GTPases by a *Toxoplasma gondii*-secreted kinase promotes macrophage survival and virulence. Cell Host Microbe.

[14] Steinfeldt T, Konen-Waisman S, Tong L, Pawlowski N, Lamkemeyer T, Sibley LD (2010). Phosphorylation of mouse immunity-related GTPase (IRG) resistance proteins is an evasion strategy for virulent *Toxoplasma gondii*. PLoS Biol.

[15] Sun X, Guan Y, Li F, Li X, Wang X, Guan Z (2012). Effects of rat cytomegalovirus on the nervous system of the early rat embryo. Virol Sin.

[16] D'Aiuto L, Di Maio R, Heath B, Raimondi G, Milosevic J, Watson AM (2012). Human induced pluripotent stem cell-derived models to investigate human cytomegalovirus infection in neural cells. PLoS One.

[17] Mutnal MB, Cheeran MC, Hu S, Lokensgard JR (2011). Murine cytomegalovirus infection of neural stem cells alters neurogenesis in the developing brain. PLoS One.

[18] Zhou J, Gan X, Wang Y, Zhang X, Ding X, Chen L, et al. *Toxoplasma gondii* prevalent in China induce weaker apoptosis of neural stem cells C17.2 via endoplasmic reticulum stress (ERS) signaling pathways. Parasit Vectors. 2015;8:73. 10.1186/s13071-015-0670-3 PMC4322664 25649541

[19] Wang T, Zhou J, Gan X, Wang H, Ding X, Chen L, et al. *Toxoplasma gondii* induces apoptosis of neural stem cells via endoplasmic reticulum stress pathway. Parasitology. 2014;141(7):988–95. 10.1017/S0031182014000183 24612639

[20] Gan X, Zhang X, Cheng Z, Chen L, Ding X, Du J (2016). *Toxoplasma gondii* inhibits differentiation of C17.2 neural stem cells through Wnt/beta-catenin signaling pathway. Biochem Biophys Res Commun.

[21] Michaelidis TM, Lie DC (2008). Wnt signaling and neural stem cells: caught in the Wnt web. Cell Tissue Res.

[22] Rocha RA, Gimeno-Alcaniz JV, Martin-Ibanez R, Canals JM, Velez D, Devesa V (2011). Arsenic and fluoride induce neural progenitor cell apoptosis. Toxicol Lett.

[23] Doering LC, Snyder EY (2000). Cholinergic expression by a neural stem cell line grafted to the adult medial septum/diagonal band complex. J Neurosci Res.

[24] Ehrt S, Schnappinger D, Casali N, Preston A, Totowa NJ (2003). Isolation of plasmids from *E. coli* by alkaline lysis. *E coli* plasmid vectors: methods and applications.

[25] LaBarre DD, Lowy RJ (2001). Improvements in methods for calculating virus titer estimates from TCID50 and plaque assays. J Virol Methods.

[26] Khaminets A, Hunn JP, Konen-Waisman S, Zhao YO, Preukschat D, Coers J (2010). Coordinated loading of IRG resistance GTPases on to the *Toxoplasma gondii* parasitophorous vacuole. Cell Microbiol.

[27] Howard JC, Hunn JP, Steinfeldt T. The IRG protein-based resistance mechanism in mice and its relation to virulence in *Toxoplasma gondii*. Curr Opin Microbiol. 2011;14(4):414–21. 10.1016/j.mib.2011.07.002 21783405

[28] Zhang X, Gan X, Cheng Z, Yu L (2016). Construction of the *Toxoplasma* ROP18 recombinant adenovirus vector and of its effect on the apoptosis of C17.2 stem cells. Chin J Zoonoses.

[29] Wan L, Gong L, Wang W, An R, Zheng M, Jiang Z, et al. *T. gondii* rhoptry protein ROP18 induces apoptosis of neural cells via endoplasmic reticulum stress pathway. Parasit Vectors. 2015;8:554. 10.1186/s13071-015-1103-z PMC4618732 26489755

[30] Van Camp JK, Beckers S, Zegers D, Van Hul W (2014). Wnt signaling and the control of human stem cell fate. Stem Cell Rev.

[31] Hari L, Brault V, Kleber M, Lee HY, Ille F, Leimeroth R (2002). Lineage-specific requirements of beta-catenin in neural crest development. J Cell Biol.

[32] Brault V, Moore R, Kutsch S, Ishibashi M, Rowitch DH, McMahon AP (2001). Inactivation of the beta-catenin gene by Wnt1-Cre-mediated deletion results in dramatic brain malformation and failure of craniofacial development. Development.

[33] Zechner D, Fujita Y, Hulsken J, Muller T, Walther I, Taketo MM, et al. Beta-catenin signals regulate cell growth and the balance between progenitor cell expansion and differentiation in the nervous system. Dev Biol. 2003;258(2):406–18. 10.1016/s0012-1606(03)00123-4 12798297

[34] Hirabayashi Y, Itoh Y, Tabata H, Nakajima K, Akiyama T, Masuyama N, Gotoh Y (2004). The Wnt/beta-catenin pathway directs neuronal differentiation of cortical neural precursor cells. Development.

[35] Massari ME, Murre C (2000). Helix-Loop-Helix proteins: regulators of transcription in eucaryotic organisms. Mol Cell Biol.

[36] Schuurmans C, Guillemot F (2002). Molecular mechanisms underlying cell fate specification in the developing telencephalon. Curr Opin Neurobiol.

